# The importance of rapid assessment tools in evaluating mental health in emergency departments among patients with chronic diseases

**DOI:** 10.3389/fpubh.2024.1258749

**Published:** 2024-03-01

**Authors:** Aditya Lal Vallath, Barath Prashanth Sivasubramanian, Diviya Bharathi Ravikumar, Akshita Lalendran, Suhasini Krishnan, Sudeshna Samanta, Snigda Banerjee, Tania Das, Ritwick Kundu, Vyom Richharia, Ravisha More, Mishika Khithani, Sahana Nazimudeen, Sasidhar Gunturu, Indraneel Dasgupta

**Affiliations:** ^1^Department of Emergency Medicine, Peerless Hospital and BK Roy Research Center, Kolkata, India; ^2^Department of Infectious Diseases, University of Texas Health Science Center at San Antonio, San Antonio, TX, United States; ^3^Department of Internal Medicine, Employees State Insurance Corporation, Chennai, Tamil Nadu, India; ^4^Department of Psychiatry, Icahn School of Medicine at Mount Sinai, New York, NY, United States; ^5^Dubai Academic Health Corporation, Dubai, United Arab Emirates; ^6^BBA Hospital Management, George Group of Colleges, Kolkata, India; ^7^Clinical Pharmacology and Research, Peerless Hospital and BK Roy Research Center, Kolkata, India; ^8^Department of Orthopedics and Trauma, Peerless Hospital and BK Roy Research Center, Kolkata, India; ^9^Department of Public Health, Bharati Vidyapeeth Deemed University Medical College, Pune, India; ^10^National AIDS Research Center, University of Aberdeen, Aberdeen, Scotland, United Kingdom; ^11^Independent Practice, Dubai, United Arab Emirates

**Keywords:** mental health, CLOX-1, six-item screener, WHO well-being index, emergency department

## Abstract

**Background:**

Rapid screening tools such as the WHO well-being Index (WWBI), Six-item screener (SIS), and the CLOX-1 test can be used to assess overall mental health and cognition, respectively. We sought to evaluate mental health with cognition in individuals with chronic diseases and stable vital signs presenting to the Emergency Department (ED).

**Methods:**

An observational study in the ED with 279 participants was conducted.

**Results:**

Chronic diseases were more prevalent among 51–70 years (43.4%) and diabetes was most common (58.8%). Fever (22.6%) and GI bleeding (32.6%) presentation were high. Participants with low WWBI had low SIS compared to the ones with higher scores (83.3% vs. 17.7%, *p* < 0.001) and also had low CLOX-1 compared to ones with high CLOX-1 (67.3% vs. 5%, <0.001). A positive correlation between WWBI with SIS (correlation coefficient = 0.305, *p* < 0.001) and CLOX-1 (0.441, <0.001). Regression analysis indicates a positive association between WWBI and the SIS (standardized regression coefficient = 0.187, 95%CI = 0.236–1.426, and *p* = 0.006) and CLOX 1 (0.338, 0.2–0.463, <0.001).

**Conclusion:**

In the ED, the evaluation of mental health even among cognitive impaired is feasible and crucial.

## Introduction

Mental health encompasses biological, psychological, and social factors that contribute to an individual’s mental state and ability to function in the environment. Additionally, it can be defined as the absence of mental illness (1). Mental illnesses are marked by clinically significant disruptions in an individual’s cognitive, emotional, or behavioral processes, often resulting in distress or impairment in daily functioning. Some examples include disorders such as major depressive disorder, certain personality disorders, posttraumatic stress disorder, and sometimes substance use disorders are all mental illnesses (2). CDC data suggests that 6 in 10 adults suffer from chronic diseases and 4 in 10 adults suffer from 2 or more diseases; other studies suggest higher incidence in adults (3, 4). In India, approximately 21% of the older adult population is estimated to have at least one chronic disease. The prevalence is higher in urban areas (29%) compared to rural areas (17%). Hypertension and diabetes account for approximately 68% of all chronic diseases among the older adults in India (5). Studies have also noted the prevalence of poor mental health among older people is high, with women, widowed/separated individuals, rural residents, those with low income, poor diet, lack of physical exercise, and multi-morbidity being at increased risk. Additionally, individuals with certain chronic diseases, such as anemia, diabetes, hyperlipidemia, cataract/glaucoma, ischemic heart disease, cerebrovascular diseases, nasopharyngitis, chronic gastroenteritis/peptic ulcer, liver diseases, cholecystitis/gallstone, arthritis, or chronic low back pain, are more likely to have poor mental health (6). Poor mental health in patients with chronic diseases is also associated with substantially higher health care costs (7). Likewise, mental illnesses are usually underdiagnosed in patients with chronic diseases due to symptom overlap (8). WHO well-being index is a rapid diagnostic tool to assess the well-being of patients and in some cases can also serve to guide the diagnosis of depression (9, 10). The applicability of rapid tools to evaluate the mental health of a patient in the Emergency department can aid in better outcomes for patients (11, 12).

As per a recent national survey by the Ministry of Health and Family Welfare Government of India, there exists a large burden of individuals with undiagnosed mental health illnesses. This not only exacerbates healthcare costs but also affects the disability in all three domains of work, social, and family life. Recommendations from the national survey include global screening for all adult patients at all points of care to be a standard of practice (13). The Emergency Department has long been considered the front door to the hospital as well as the safety net for the population as all forms of health care can be provided irrespective of symptom acuity/criticality, demographic factors, etc. It has long been known that the patients seen in a large tertiary care emergency department will accurately portray the patient population seen in the surrounding community. It is with that in mind many studies in the ED have been conducted to screen patients for mental health illness (14, 15).

We aimed to evaluate the mental health status of hemodynamically stable individuals with chronic diseases who presented to the ED after they had had relief from their presenting complaints. We sought to evaluate the association of cognition function with mental health status. By investigating the association between mental health status (with the WHO well-being index) and cognition (Six Item Scanner and CLOX-1 clock drawing test), in individuals with chronic diseases who visited the ED in those with stable vital signs, we would be able to contribute to the knowledge of mental health, cognitive function, and their associations in individuals with chronic diseases seeking care in the ED.

## Methodology

### Study setting and design

A population-based descriptive observational study was conducted in the Emergency Department of Peerless Hospital Kolkata, India, to gather data on its operations. A nonprobabilistic convenience sampling technique was employed and the study was conducted from November 2022 to February 2023, allowing for a comprehensive analysis of the ED’s operations using the selected sampling technique.

### The population involved

The study focused on patients who presented with chronic diseases to the ED, which is defined as illnesses lasting more than 1 year, irrespective of medication compliance. The population included those with stable vitals, over 18 years of age, diagnosed with a chronic illness, and without psychiatric disorders. Once informed consent was obtained, they were required to be able to complete the survey tasks. Patients were only allowed to participate in the study after they were relieved of their presenting complaints or they achieved symptomatic relief. This was important as symptoms such as pain and fever can affect cognition as well as exacerbate mood (16). Patients who did not meet these criteria, including those with unstable vitals, under 18 years of age, or with an illness less than 1 year in duration, were excluded. Patients with known cognitive impairment, a history of psychiatric illness, or those unable to complete the survey tasks for any reason were also not eligible.

### Sample size

Based on the study by Ingle et al. which showed a prevalence of depression at 25%, a sample size of 300 was calculated (17). However, the actual sample size for our study is 297.

### Procedure and scales used

To collect information from the participants, a pretested semi-structured questionnaire consisting of four items was used in this study. The questionnaire initially included the Six-Item Screener and CLOX-1 to identify cognitive impairment. Patients who scored below the threshold for cognitive impairment were administered the WHO-5 Well-Being Index. The WHO 5 Well-being Index has been used globally and its psychometric properties have been validated in multiple studies (18–20). One particular study conducted in Bengali, in rural West Bengal and Odisha (the native language of patients in the ED) showed a validation of *r* = 0.542, *p* < 0.01 (21–23). Test–retest reliability of the scale was previously reported to be *r* = 0.713 (21). The scale, previously measured in an Indian population of those 60 years and above, has reported sensitivity of 97.0% and specificity of 86.4%, with an overall accuracy of 0.89 (22). In another study by Topp et al., (9) the WWBI was found to be an adequate screening tool as it demonstrated high sensitivity (98%) and specificity (83%). The WHO-5 questionnaire is useful in the assessment of depression, suicidality, stress, addiction, illicit substance usage, and in many organic disorders (cardiovascular diseases, Parkinson’s disease, and chronic pain) (10, 24). The consistent application of the WHO-5 across diverse research areas demonstrates its versatility and validity as a measure of psychological well-being (10, 25). No investigations were performed during the study. The interviews were conducted in the patient’s native language, and strict patient confidentiality was maintained throughout the study. Information about the patients’ chronic illnesses was obtained from the patients. The study utilized several variables, including personal history, disease details, the reason for ED visits, and vital signs such as blood pressure, heart rate, and respiratory rate.

### Statistical portion

Data was collected, cross-checked, and analyzed using SPSS software using appropriate statistical tests. All continuous variables were represented as Mean and Standard Deviation. Qualitative data were expressed as frequencies and percentages. The chi-square test was used to compare categorical variables. Linear regression analysis was used to identify associations between the scales. Spearman correlation was used to assess the correlation of the data. A value of *p* of ≤0.05 was considered statistically significant.

### Ethical consideration

This study has been approved by the ethics committee with the reference number (REF PHH/SC/ER/3184/2022). Written informed consent was obtained from all participants, which included consent to the collection, analysis, and publication of relevant findings. The privacy and confidentiality of the participants were protected throughout the study, and all data collected were de-identified to maintain participant anonymity. No funding was used for this study. These measures were taken to ensure the ethical conduct of the study and to protect the privacy and confidentiality of the participants involved.

## Results

Of 297 participants, 18 participants did not complete the WHO well-being index or did not want to further participate in the study. Among 279 participants, chronic diseases were more prevalent among 51–70 years (121, 43.4%) and females (147, 52.7%). We found 237 (84.9%), 178 (63.8%), and 202 (72.4%) had high scores on cognition (SIS and CLOX-1), and mental health (WHO well-being index) respectively (Table 1).

**Table 1:** Shows the association of different age groups, gender, chronic diseases and reasons for emergency department visits with the 6-item screener, CLOX-1 scale, and WHO well-being index (Kolkata, India, 2023).

Age groups		Six item screener		CLOX-1 score		WHO well-being index		
		<4 (*n* = 42)	4–6 (*n* = 237)	<10 (*n* = 101)	10–15 (*n* = 178)	<13 (*n* = 77)	>13 (*n* = 202)	Total (*n* = 279)
18–30 years		0	23	0	23	0	23	23
31–50 years		0	68	13	55	4	64	68
51–70 years		19	102	34	87	36	85	121
71–90 years		23	44	54	13	37	30	67
Total *n*		42	237	101	178	77	202	279
Gender		<4 (*n* = 42)	4–6 (*n* = 237)	<10 (*n* = 101)	10–15 (*n* = 178)	<13 (*n* = 77)	>13 (*n* = 202)	
Male (*n* = 132)		11***	121***	32****	100****	18*****	114*****	
Female (*n* = 147)		31***	116***	69****	78****	59*****	88*****	
Total		42***	237***	101****	178****	77*****	202*****	
Primary complaint		<4 (*n* = 42)	4–6 (*n* = 237)	<10 (*n* = 101)	10–15 (*n* = 178)	<13 (*n* = 77)	>13 (*n* = 202)	Total (*n* = 279)
Cardiorespiratory complaints		14	18	25	7	17	15	32
Abdominal pain, renal and urinary complaints		0	46	7	39	8	38	46
Neurological complaints and dizziness		10	37	20	27	15	32	47
Fever		13	50	23	40	26	37	63
Gastrointestinal bleeding		5	86	26	65	11	80	91
Chronic disease/ Comorbidities		<4 (*n* = 42)	4–6 (*n* = 237)	<10 (*n* = 101)	10–15 (*n* = 178)	<13 (*n* = 77)	>13 (*n* = 202)	Total (*n* = 297)
Diabetes	No	16	99	35	80	22	93	115
	Yes	26	138	66	98	55	109	164
Hypertension	No	19	103	44	78	29	93	122
	Yes	23	134	57	100	48	109	157
Thyroid disease	No	32	169	78	123	61	140	201
	Yes	10	68	23	55	16	62	78
Heart disease*	No	36	215	84	167	65	186	251
	Yes	6	22	17	11	12	16	28
Chronic kidney disease	No	38	226	91	173	67	197	264
	Yes	4	11	10	5	10	5	15
Lung disease	No	37	219	90	166	70	186	256
	Yes	5	18	11	12	7	16	23
Liver and Gall Bladder disease	No	42	225	101	166	77	190	267
	Yes	0	12	0	12	0	12	12
Other Causes**	No	39	225	98	166	77	187	264
	Yes	3	12	3	12	0	15	15

*CAD, Coronary artery diseases.

*CHF, Congestive heart failure.

**Other causes, Pancreas, Fractures, Rheumatoid Arthritis.

*** Chi-square value of 8.848, value of *p* = 0.003.

****Chi-square value of 15.512, value of *p* < 0.001.

*****Chi-square value of 24.442, value of *p* < 0.001.

All of the participants 18 to 30 years of age (23, 9.7%) had high scores on cognition (SIS and CLOX-1) and mental health. The majority of the participants were of 51–70 years of age which was 121 (43.4%) and among these, 102 (84.3%), 87 (71.9%), and 85 (70.2%) participants had high scores on cognition (SIS and CLOX-1) and mental health, respectively. Females were predominant in this study 147 (52.7%), however, more females had lower scores on SIS (73.8% vs. 26.2%, value of *p* = 0.003), CLOX-1 (68.3% vs. 31.7%, <0.001), and mental health (76.6% vs. 23.4%, <0.001) when compared to males. 164 (58.8%) participants had diabetes and among these, 138 (84.1%, *p* = 0.655), 98 (59.8%, 0.093), and 109 (66.5%, 0.008) had high scores on cognition (SIS and CLOX-1) and mental health, respectively. We found 157 (56.3%) participants had hypertension and among these, 134 (85.4%, 0.83), 100 (63.7%, 0.967), and 109 (69.4%, 0.207) had high scores on cognition (SIS and CLOX-1) and mental health, respectively. Fever (63, 22.6%) and gastrointestinal bleeding (91, 32.6%) were the main complaints. Among those 63 with fever, 50 (79.4%), 40 (63.5%), and 37 (58.7%) had high scores on cognition (SIS and CLOX-1) and mental health, respectively. Among 91 with gastrointestinal bleeding, 86 (94.5%), 65 (71.4%), and 80 (87.9%) had high scores on cognition (SIS and CLOX-1) and mental health, respectively (Table 2).

**Table 2:** Shows the vital signs of the participants measured at the emergency department (Kolkata, India, 2023).

	Temperature in F	SBP	DBP	Pulse	Respiratory rate	SPO2
Number of participants	297	297	297	297	297	297
Median	98	140	80	87	18	99

Our results showed 164 (58.8%) participants had diabetes and among these, 138 (84.1%, *p* = 0.655), 98 (59.8%, 0.093), and 109 (66.5%, 0.008) had high scores on cognition (SIS and CLOX-1) and mental health, respectively. We found 157 (56.3%) participants had hypertension and among these, 134 (85.4%, 0.83), 100 (63.7%, 0.967), and 109 (69.4%, 0.207) had high scores on cognition (SIS and CLOX-1) and mental health, respectively. Most participants presented to ED with fever (63, 22.6%) and gastrointestinal bleeding (91, 32.6%) as the main complaint. Among the 63 with fever, 50 (79.4%), 40 (63.5%), and 37 (58.7%) had high scores on cognition (SIS and CLOX-1), and mental health, respectively. Among the 91 with gastrointestinal bleeding, 86 (94.5%), 65 (71.4%), and 80 (87.9%) had high scores on cognition (SIS and CLOX-1) and mental health, respectively.

Our analysis revealed participants with low WHO well-being scores were more among those with low 6-item screener scores compared to the ones with higher scores (83.3% vs. 17.7%, *p* < 0.001). Likewise, those with low WHO well-being scores were more in those with low CLOX-1 scores compared to ones with high CLOX-1 scores (67.3% vs. 5%, <0.001) Spearman correlation revealed a positive correlation was found between the WHO well-being index with the 6-item screener (correlation coefficient = 0.305, *p* < 0.001) and CLOX-1 scores (0.441, <0.001). Linear regression analysis was performed with the 6-item screening score and CLOX 1 scores as the independent variable and the WHO well-being index score as the dependent variable. The standardized regression coefficient for 6-item screening was 0.187, with a standard error of 0.302, 95%CI = 0.236 to 1.426, and a value of *p* of 0.006. This indicates a positive association between mental health and cognition. The standardized regression coefficient for CLOX 1 was 0.338, with a standard error of 0.067, 0.2 to 0.463, <0.001, which indicates a positive association between them (Table 3).

**Table 3:** Association between WHO well-being index with the 6-item screener and CLOX-1 (Kolkata, India, 2023).

		WHO well-being index				
		<13	>13	Total	Chi-square	value of *p*
Six item screener	<4	35	7	42	76.864	<0.001
	4–6	42	195	237		
	Total	77	202	279		
CLOX-1	<10	68	33	101	125.046	<0.001
	>13	9	169	178		
	Total	77	202	279		

Participants with low WWBI scores were more among those with low SIS scores compared to the ones with higher scores (83.3% vs. 17.7%, *p* < 0.001). Likewise, those with low WWBI scores were more in those with low CLOX-1 scores than those with high CLOX-1 scores (67.3% vs. 5%, <0.001) (Table 4).

**Table 4:** Linear regression assessing the association between WHO well-being index with the 6-item screener and CLOX-1 (Kolkata, India, 2023).

		Standardized coefficients	value of *p*	95% Confidence interval	
	Std. error	Beta		Lower bound	Upper bound
Six item screener	0.302	0.187	0.006	0.236	1.426
CLOX-1 score	0.067	0.338	<0.001	0.2	0.463

Linear regression analysis was performed with the 6 item screening score and CLOX 1 scores as the independent variable and the WHO well-being index score as the dependent variable. The standardized regression coefficient for 6-item screening was 0.187, with a standard error of 0.302, 95%CI = 0.236 to 1.426, and a value of p of 0.006. This indicates a positive association between mental health and cognition. The standardized regression coefficient for CLOX 1 was 0.338, with a standard error of 0.067, 0.2 to 0.463, <0.001, which indicates a positive association between them.

## Discussion

The use of rapid assessment tools such as the WWBI, Six-item screener (SIS), and CLOX-1 in EDs has been gaining attention for their potential to improve patient outcomes through assessing psychological well-being and cognition (26, 27). Older adults individuals have cardiovascular diseases, particularly diabetes, and hypertension, as well as thyroid disorders and asthma as co-morbidities, and this in turn leads to a decline in cognition (2, 3, 6, 8, 24, 28–33). However, literature comparing the three tests and identifying positive associations between them is scarce. To cover this, we sought to evaluate the mental health status of hemodynamically stable individuals with chronic diseases who presented to the ED. We also evaluated the association of cognition function with mental health status. The study found significant association and positive correlation between WWBI with SIS and CLOX-1 scores. Regression analysis showed a positive association between WWBI and both SIS and CLOX-1 scores. This suggests that these rapid assessment tools can provide valuable information on a patient’s overall health status which can lead to better-informed treatment decisions. The link between chronic diseases, poor mental health, and cognitive function has been explored vastly.

In a literature review by Topp et al., the WWBI was found to be an adequate screening tool for depression and it demonstrated high sensitivity (98%) and specificity (83%) (9). In a study comprising 274 participants, 73% were female, and a WWBI mean score of 14.32 was reported, indicating poor well-being. These findings were similar to our study’s results, indicating unaddressed issues in females (76.6% vs. 23.4%, <0.001) (34). The SIS derived from the MMSE is capable of assessing cognitive impairment with sensitivity of 63% (35). We found a positive correlation between the SIS and mental health (correlation coefficient = 0.305, *p* < 0.001). The CLOX-1 test is a performance test that requires the patient to draw a clock face with all of the numbers and to set the hands to a specific time. Royall et al. demonstrated CLOX-1 and found it to be a strong indicator of cognitive function based on a high correlation with EXIT 25 (*r* = −0.83) (36). The SIS is administered verbally and disregards the need for equipment, thereby saving time, but does not consider potential language barriers that may arise, especially in a multiethnic population (27). The CLOX-1 test requires the use of pencil and paper and has decreased vulnerability to language and/or education bias (36). Hirschman et al. showed a difference in identifying cognitive impairment between the SIS model (42%) and the CLOX 1 (36%) in an ED setting (37). Our results linked to a positive association between those with low WWBI scores and low CLOX-1 scores (correlation coefficient = 0.441, *p* < 0.001), indicating that those with decreased well-being had impaired cognitive function. Callahan et al. support our findings that patients with impaired cognitive findings have poor mental well being (38).

Patients with chronic diseases often have problems with processing emotions and are more likely to experience stressful events. These significantly impact the quality of life (39). Novel multidimensional approaches for chronic disease management reported by Dan Ling et al. include depression screening, care planning, and health coaching, to support self-management of hypertension (33). A tailor-made interdisciplinary management approach for each patient, as well as conducting more high-quality research on the topic. By addressing the psychological and psychopathological factors that contribute to chronic diseases, we can improve the quality of life and subjective well-being of patients with these conditions.

## Limitations

This study is novel, the first study to implement health screening in the ED in Eastern India, using the three rapid assessment tools. This study has some limitations. The screening methods can be applied only after triaging is done, in the absence of any previous medical records, and with the support of adequate laboratory testing. In some instances, patients may require a caregiver to be present to complete screening. All points considered, it could mean a time-consuming process. More efficient screening criteria that cover all cognitive domains should be designed to improve patient outcomes. Our small sample size could potentially limit the generalizability of our findings. In our study, we were able to identify a weak but positive correlation between the two cognition scales and the well being index. Further research is needed to develop a rapid testing tool that covers all the domains of cognition and can be administered in the ED setting to assess mental health.

## Conclusion

The use of rapid assessment tools such as the WWBI, SIS, and CLOX-1 in ED can aid in better patient outcomes. The study conducted in a tertiary care hospital in India found that there is a positive association between these tools concerning cognitive function, functional status, and overall well-being among patients with chronic diseases. These findings highlight the importance of evaluating mental health in individuals presenting with cognitive impairment at EDs. Early detection and management of these conditions are crucial for improving outcomes for affected individuals while reducing healthcare costs associated with advanced stages of mental illness.

## Data availability statement

The original contributions presented in the study are included in the article/supplementary material, further inquiries can be directed to the corresponding author.

## Ethics statement

The studies involving humans were approved by Drugs Controller General of India - ECR/232/INST/WB/2013/RR-19. The studies were conducted in accordance with the local legislation and institutional requirements. The participants provided their written informed consent to participate in this study.

## Author contributions

AV: Conceptualization, Data curation, Formal analysis, Methodology, Supervision, Writing – original draft, Writing – review & editing. BS: Data curation, Formal analysis, Supervision, Writing – original draft, Writing – review & editing. DR: Data curation, Formal analysis, Supervision, Writing – original draft, Writing – review & editing. AL: Writing – original draft. SK: Writing – original draft. SS: Data curation, Writing – original draft. SB: Writing – original draft. TD: Data curation, Writing – original draft. RK: Data curation, Writing – original draft. VR: Data curation, Writing – original draft. RM: Data curation, Writing – original draft. MK: Data curation, Writing – original draft. SN: Data curation, Writing – original draft. SG: Supervision, Writing – review & editing. ID: Supervision, Writing – review & editing.
